# Comparison of Carbapenem vs. Amikacin Antimicrobial Therapy for Pediatric Acute Pyelonephritis Caused by Extended-Spectrum Β-Lactamase-Positive Enterobacteriaceae

**DOI:** 10.3390/children12070945

**Published:** 2025-07-18

**Authors:** Burcu Ceylan Cura Yayla, Tuğba Bedir Demirdağ, Anıl Tapısız, Yeşim Özdemir Atikel, Hasan Tezer, Elif Ayça Şahin, Kayhan Çağlar, Sevcan A. Bakkaloğlu, Necla Buyan

**Affiliations:** 1 Department of Pediatric Infectious Diseases, Ankara Training and Research Hospital, University of Health Sciences, 06230 Ankara, Türkiye; 2 Department of Pediatric Infectious Diseases, Faculty of Medicine, Gazi University, 06500 Ankara, Türkiye; tugbabedir@gmail.com (T.B.D.); anilaktas@gazi.edu.tr (A.T.); hasantezer@yahoo.com (H.T.); 3 Department of Pediatric Nephrology, Eskişehir City Hospital, 26080 Eskisehir, Türkiye; yesozdemir@gmail.com; 4 Department of Microbiology, Faculty of Medicine, Gazi University, 06500 Ankara, Türkiye; elifaycasahin@gazi.edu.tr (E.A.Ş.); kcaglar@gazi.edu.tr (K.Ç.); 5 Department of Pediatric Nephrology, Faculty of Medicine, Gazi University, 06500 Ankara, Türkiye; sevcan@gazi.edu.tr (S.A.B.); nbuyan@gazi.edu.tr (N.B.)

**Keywords:** pediatric acute pyelonephritis, extended-spectrum β-lactamase, Enterobacteriaceae, amikacin, carbapenem

## Abstract

**Objectives**: Acute pyelonephritis (APN) caused by extended-spectrum β-lactamase (ESBL)-positive Enterobacteriaceae poses a growing therapeutic challenge in children, as carbapenems remain the mainstay of treatment even when susceptibility to alternative agents such as amikacin is demonstrated. However, the widespread and inappropriate use of carbapenems can lead to carbapenem resistance. The aim of this study was to compare the clinical efficacy of amikacin and carbapenems in the management of pediatric acute pyelonephritis caused by ESBL-positive Enterobacteriaceae. **Methods**: We analyzed cases of pediatric acute pyelonephritis caused by ESBL-positive Enterobacteriaceae that were treated with either carbapenems or amikacin over a two-year period. This study compared microbiological cure, clinical improvement, and recurrence rates across the amikacin and carbapenem treatment groups. **Results**: Fifty-five patients were evaluated. The median age of the patients was 3 years (range, 0.1–13 years). The causative agents were *E. coli* in 43 cases (78.2%) and *Klebsiella* spp. in 12 cases (21.8%). All were susceptible to both carbapenem and amikacin in vitro. Twenty patients (36.3%) received a carbapenem and thirty-five (63.7%) received amikacin. Twenty-four (43.6%) had an underlying urological disease. No difference was observed between the groups in terms of microbiological cure, clinical improvement, or recurrence rates. **Conclusions**: Amikacin may be a potential alternative to carbapenems for treating pediatric ESBL-positive APN when in vitro susceptibility is confirmed.

## 1. Introduction

Urinary tract infections (UTIs) are among the most frequent infectious diseases in children and are a major cause of hospitalization. They can result in long-term morbidities such as renal scarring, hypertension, and end-stage renal failure [1]. UTIs are defined as an infection (typically bacterial) in any part of the urinary tract, accompanied by associated signs and symptoms. These infections range from cystitis, which presents with lower urinary tract symptoms, to acute pyelonephritis (APN), characterized by systemic and abdominal symptoms [2].

Several factors, including age, gender, and circumcision status, affect the incidence of UTIs in pediatrics. In febrile infants, the estimated prevalence is around 7%. The prevalence of UTIs is notably higher in uncircumcised male infants, especially in the first three months. Girls experience UTIs two to four times more frequently than circumcised boys. Among older children presenting with fever and/or urinary symptoms, the reported prevalence of UTIs is approximately 7.8% [3]. By the age of seven, it is estimated that around 8% of girls and 2% of boys will have had at least one episode of UTI [4].

In the pediatric population, *E. coli* remains the predominant uropathogen, with reported prevalence rates ranging between 80% and 90% across different studies [5,6]. In addition to *E. coli,* other Gram-negative bacteria such as *Klebsiella*, *Proteus*, *Enterobacter*, and *Citrobacter* are also responsible for UTIs in children [5]. Among Gram-positive organisms, *Staphylococcus saprophyticus*, *Enterococcus*, and, less frequently, *Staphylococcus aureus* may be implicated [7]. The antibiotic susceptibility of Gram-negative bacilli has shifted over time, and the prevalence of extended-spectrum β-lactamase (ESBL)-positive species is rising [8,9]. The rate of UTIs caused by ESBL-positive organisms has shown a global rise, currently estimated at 7.4% in North America, 18.8% in Europe, 27.7% in South Asia, and 60% in India [10,11,12,13].

The treatment of ESBL-positive bacterial infections may be difficult. Cefepime, aminoglycosides, carbapenems, trimethoprim–sulfamethoxazole, and quinolones are generally effective and can be considered for empirical therapy [14].

Due to their broad-spectrum activity, favorable pharmacokinetic properties, and consistent therapeutic efficacy, carbapenems are considered the preferred agents for treating severe infections caused by ESBL-positive bacteria [14,15]. The overuse of carbapenems has led to the alarming rise in carbapenem-resistant Enterobacteriaceae, now recognized as a critical concern in the context of global antimicrobial resistance [15,16]. This highlights the need for rational antimicrobial use and the exploration of effective, narrower-spectrum alternatives.

Amikacin demonstrates high in vitro susceptibility rates against ESBL-positive *Enterobacteriaceae* and offers several pharmacokinetic advantages, including a narrow antibacterial spectrum and the convenience of once-daily dosing [17,18]. Its ability to achieve high urinary concentrations makes it especially effective in the treatment of UTIs. Therefore, several guidelines and recent studies support the use of aminoglycosides, including amikacin, as monotherapy in selected pediatric UTIs when antimicrobial susceptibility is confirmed [17,18].

To reduce the risk of developing resistance, carbapenems should be avoided in empirical therapy when possible and reserved as a last-line treatment. Despite increasing awareness of this issue, well-designed outcome studies comparing carbapenems with alternative agents such as amikacin in pediatric populations remain scarce. The objective of this study was to evaluate and compare the clinical effectiveness and safety of carbapenem and amikacin in managing pediatric acute pyelonephritis due to ESBL-positive Enterobacteriaceae, with the goal of supporting evidence-based treatment decisions.

## 2. Materials and Methods

### 2.1. Study Design

This retrospective study was conducted in the Pediatric Infectious Diseases and Pediatric Nephrology departments of a pediatric tertiary care unit. Children aged between 1 month and 18 years diagnosed with APN caused by ESBL-positive Gram-negative bacilli over two consecutive years were evaluated retrospectively. The analysis was based on information retrieved from medical records, including the following variables: age, sex, unit, presence of comorbid disease, duration of hospitalization, presence of urological disease, presence of a urinary catheter, antibiotic use within the last six months (for any reason), prior hospitalization with the same diagnosis, blood test results (renal function tests, acute phase reactants, and blood count), microbiologic data, and antibiotic susceptibility results. Additionally, clinical improvement, microbiological cure, and recurrence of UTI were noted based on parameters such as urine culture results, time to fever reduction, and resolution of pyuria. Exclusion criteria were being younger than one month of age, hospitalized with a diagnosis of cystitis, infected with non-ESBL-positive bacteria, having an immunosuppressive condition, and permanent urinary catheterization.

The patients were treated with either intramuscular or intravenous amikacin (15 mg/kg/d) or intravenous carbapenem. In the carbapenem group, patients received either intravenous meropenem (60 mg/kg/d) or intravenous ertapenem (30 mg/kg/d). Intramuscular or intravenous amikacin treatment was decided according to the patient’s hospitalization status. Hospital admission was considered necessary in cases presenting with clinical signs of urosepsis (such as toxic appearance, hypotension, or poor peripheral perfusion), persistent vomiting, an inability to tolerate oral medications, insufficient outpatient follow-up, or a lack of clinical improvement under outpatient treatment [19]. Rates of microbiological cure, clinical improvement, and recurrence were evaluated in both groups.

The guideline-based treatment protocol applied in our hospital is as follows: When a patient presents with UTI-related symptoms, empirical ceftriaxone (75 mg/kg/d) or amikacin (15 mg/kg/d) treatment is initiated based on the results of urinalysis. After obtaining antimicrobial susceptibility test results, patients with ESBL-positive Gram-negative bacilli who were receiving ceftriaxone were switched to amikacin or a carbapenem. In the treatment of pediatric febrile UTIs, aminoglycosides are recommended as treatment options in the AAP guideline [19]. In this context, amikacin may be used in our clinical practice in our center for the treatment of acute pyelonephritis. Our study is retrospective, and the treatment modality was selected according to the clinician’s preference not to the severity of the patients. Antibiotic choice (carbapenem or amikacin) was not determined by a predefined protocol but rather reflected routine clinical decision-making practices during the study period. Patients were not stratified according to infection severity at the time of treatment initiation. Treatment was not changed for patients with ESBL-positive Gram-negative bacilli who were empirically started on amikacin.

### 2.2. Definitions and Microbiologic Identification

Transurethral catheterization was performed to obtain urine samples in children younger than 2 years of age, while midstream clean-catch samples were used for older patients. A UTI was defined as the presence of a single pathogen growing ≥50,000 colony-forming units (CFU)/mL in catheterized samples or ≥100,000 CFU/mL in midstream collections, accompanied by pyuria—defined as more than 5 white blood cells per high-power field (WBC/HPF) on urinalysis—in a symptomatic child. The diagnosis of APN was supported by systemic symptoms such as fever, malaise, nausea, vomiting, and gastrointestinal complaints like diarrhea, in addition to localized signs such as abdominal, flank, or back pain, together with the aforementioned laboratory findings [20,21].

Relapse was defined as the reappearance of UTI symptoms with a positive urine culture caused by the same pathogen within 14 days after completion of antibiotic therapy. Recurrence, on the other hand, was defined as a new episode of UTI occurring more than 14 days after treatment completion, which could be caused by the same or a different pathogen [22]. In our retrospective study, relapse and recurrence were assessed based on medical records within 6 months after discharge. These events were identified through documented outpatient visits or hospital readmissions due to UTIs.

Clinical improvement was defined by the resolution of fever (<37.5°C for at least 24 h), an improvement in urinary symptoms, the resolution of pyuria (≤5 WBC/HPF on follow-up urinalysis), and a decrease in serum inflammatory markers within 72 h [20].

Microbiological cure was defined as a negative urine culture obtained 48–72 h after the initiation of therapy, with samples collected via transurethral bladder catheterization younger than 2 years of age and by midstream clean-catch collection in older children [20].

This research included 55 isolates in the Enterobacteriaceae family (*E. coli*, *Klebsiella pneumoniae*), with ESBL production detected using the microdilution method in VITEK 2/AES (ASTGN27 card; bioMérieux, Marcy l’Étoile, France) according to the procedure specified in the manufacturer’s instructions [23].

Antimicrobial susceptibility testing was also performed by VITEK 2/AES (ASTGN27 card, bioMérieux, Marcy l’Étoile, France) according to the manufacturer’s instructions and the criteria of European Committee of Antimicrobial Susceptibility. Bacterial identification was performed by matrix-assisted laser desorption ionization–time of flight (MALDI-TOF) mass spectrometry (bioMérieux, France) [23].

### 2.3. Ethical Approval

This study was conducted with the approval of the Ethical Committee, Faculty of Medicine, Gazi University (dated 2 March 2022, number 127).

### 2.4. Statistical Analyses

Patient information was retrospectively extracted from hospital medical records. Statistical analyses were performed using IBM SPSS Statistics software, version 22 (IBM Corp., Armonk, NY, USA). Continuous variables with normal distribution are expressed as the mean ± standard deviation and were analyzed using the independent-samples Student’s *t*-test. Non-normally distributed continuous data are presented as the median with range and were compared using the Mann–Whitney *U* test. Categorical variables are described as frequencies and percentages, and group comparisons were made using the chi-square test or Fisher’s exact test, where appropriate.

Due to the retrospective nature of this study, data analysis was not performed in a blinded manner.

## 3. Results

The study population consisted of fifty-five pediatric patients. Thirty-five (63.6%) of these patients were female. The median age was 3 years (0.1–13.0 years). Thirty-two patients (58.2%) were hospitalized. All infections were community-acquired. Twenty-four (43.6%) of the patients had an underlying urological disease, and nine (16.4%) had at least one comorbidity other than urological diseases. Twenty-eight (50.9%) patients had a history of antibiotic consumption within the last six months and twenty-five (45.5%) had a history of UTI within the preceding year. Patient characteristics/demographic data and risk factors are shown in Table 1.

At admission, the most frequent clinical symptom was fever in 51 patients (92.7%). Other symptoms included irritability in 18 (32.7%), a decreased oral intake in 11 (20.0%), vomiting in 10 (18.2%), urinary symptoms (dysuria, frequency) in 12 (21.8%), nausea in 8 (14.5%), flank pain in 4 (7.3%), and abdominal pain in 3 (5.5%). No statistically significant differences were found between the two treatment groups in terms of symptom frequencies (*p* > 0.05 for all comparisons).

**Figure 1:**
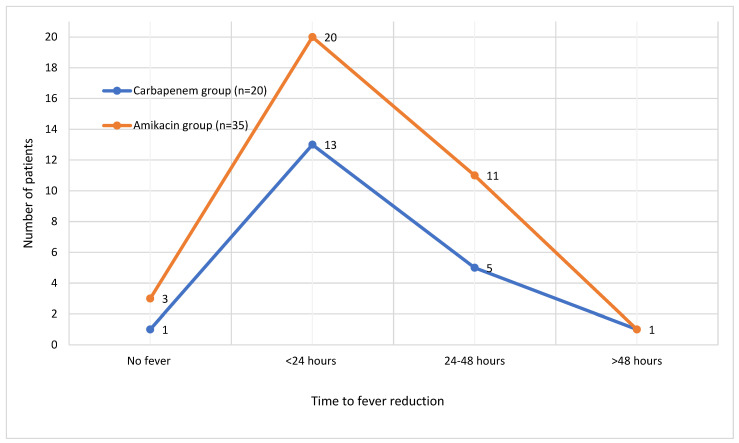
Time to fever reduction in patients.

**Figure 2:**
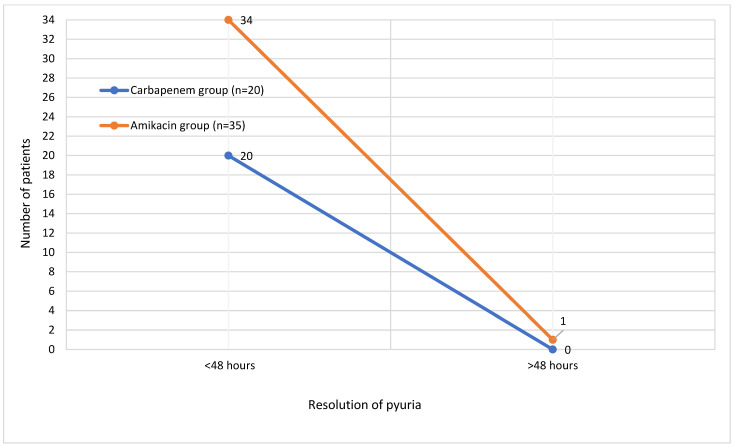
Time to resolution of pyuria in patients.

Fever resolved within 24 h in 33 patients (60%), within 24–48 h in 16 patients (29.1%), and after more than 48 h in 2 (3.6%) patients. The distribution of time to fever resolution in both treatment groups is illustrated in Figure 1. Most patients showed a resolution of pyuria within 48 h after treatment initiation. The time to resolution of pyuria in all patients is demonstrated in Figure 2. There were no significant differences between the groups in terms of time to fever reduction and resolution of pyuria (*p* > 0.05) (Table 1).

Twenty patients (36.3%) were treated with carbapenems, and thirty-five (63.7%) were treated with amikacin. There was no statistically significant difference between the carbapenem and amikacin group in terms of median age or sex distribution (*p* = 0.089, *p* = 0.895). Antibiotic use in the last six months and history of UTI in the last year did not differ between the amikacin and carbapenem group (*p* = 0.346, *p* = 0.370). There was also no statistically significant difference between the groups in the frequency underlying urological disease or comorbidities (*p* = 0.585, *p* = 0.203) (Table 1).

The etiologic agents of UTI were *E. coli* (78.2%) and *Klebsiella* spp. (21.8%). The distribution of the pathogens in the urine cultures did not differ statistically significantly between the carbapenem and amikacin groups (*p* = 0.740) (Table 1).

All of the patients were successfully treated except for one. This patient was a 2-year-old child with ureteropelvic junction stenosis. Fever and pyuria did not resolve in 72 h with amikacin therapy. After switching to meropenem therapy, she showed clinical improvement.

The renal function tests of all patients were stable during the 6-month period. Ototoxicity was not systematically assessed and therefore could not be evaluated. No relapses were observed in either group during the follow-up period.

## 4. Discussion

The findings of this study suggest that amikacin may be a promising alternative to carbapenems in the management of pediatric APN caused by ESBL-positive Enterobacteriaceae, as no significant differences in clinical outcomes were observed between the two treatment groups. Given concerns about the widespread use of carbapenems leading to resistance, our results suggest that amikacin, a narrow-spectrum antibiotic, is a viable option for pathogens with in vitro susceptibility. These findings emphasize the importance of antimicrobial stewardship in preserving the efficacy of carbapenems by encouraging their selective use and supporting the consideration of narrower-spectrum agents such as amikacin when appropriate.

The prevalence of ESBL-positive *E. coli* and *K. pneumoniae* as causative agents of UTIs is rising, even among community-acquired infections, which differ between reported cases [24]. In community-onset UTIs, Bou Chebl et al. reported the rate of ESBL-positive *E. coli* as 24.9%, while Tuzun et al. reported this rate as 50.5% [25,26].

Numerous studies have described *E. coli* and *K. pneumoniae* as predominant pathogens in ESBL-positive UTIs. For instance, Agegnehu et al. reported *E. coli* as the most frequently isolated species (44.4%), followed by *K. pneumoniae* (27.8%) [27]. In another report by Balasubramanian et al., 92.5% of ESBL-positive *Enterobacteriaceae* in UTIs were *E. coli* and 7.5% were *K. pneumoniae* [28]. Similarly to the literature, this study revealed *E. coli* (78.2%) and *Klebsiella* spp. (21.8%) as the most common pathogens of ESBL-positive UTI.

The treatment of UTIs caused by ESBL-positive *Enterobacteriaceae* in pediatric patients presents a significant clinical challenge due to the increase in antibiotic-resistance [29]. ESBL-positive UTIs increasingly require the use of broad-spectrum antibiotics in treatment, which leads to the development of resistance to potent antibiotics such as carbapenems [30]. While carbapenems continue to be essential for managing severe infections, the use of alternative antimicrobials should be considered in cases of uncomplicated UTI [24]. Although the effectiveness of non-carbapenem antibiotics in treating ESBL-associated UTIs is well established in adult populations, comparative data in children remain scarce [31].

In a study by Park et al., non-carbapenem agents, including aminoglycosides, were found to be similarly effective to carbapenems for managing community-onset acute pyelonephritis caused by ESBL-positive *E. coli*, provided that in vitro susceptibility was confirmed [29]. Similarly, Polat et al. demonstrated that once-daily intramuscular aminoglycoside administration achieved comparable outcomes to carbapenem therapy in pediatric patients treated on an outpatient basis [17]. Another report showed that aminoglycosides were found to be non-inferior to carbapenems in the management of bloodstream infections of a urinary source in terms of 30-day mortality [18]. According to this study, time to fever resolution and culture sterilization showed similar values in the aminoglycoside and carbapenem groups. There is limited clinical data in children related to the use of amikacin in the management of UTIs caused by ESBL-positive *E. coli*. Thus, the results of this study indicate a valuable contribution to the pediatric literature, as there are very few reports about aminoglycosides in the inpatient treatment of ESBL-positive *Enterobacteriaceae infections*. This study also promotes antimicrobial stewardship, highlighting the need to avoid the overuse of broad-spectrum antibiotics such as carbapenems.

Regarding the recurrence of bacteriuria, Zohar et al. was unable to demonstrate the non-inferiority of aminoglycosides in patients with bloodstream infections of a urinary source caused by ESBL-positive *Enterobacteriaceae* [18]. In another study, the rate of UTI relapse after aminoglycoside therapy was 2% [17]. In contrast to the literature, the rate of recurrent bacteriuria did not differ significantly between the aminoglycoside and carbapenem groups in our study. In other words, carbapenem was not superior to aminoglycosides in preventing UTI recurrence. Although our patient group was small, this new data is valuable in highlighting the selection of aminoglycosides in the empirical treatment of ESBL-induced pyelonephritis. The evidence cannot be generalized yet, but this finding may guide future research and should be supported by prospective, well-designed, randomized controlled pediatric studies.

Antibiotic selection is influenced by factors such as the severity of the illness, the presence of underlying conditions, and response to empirical therapy [15]. Park et al. showed that non-carbapenem antibiotics and carbapenems are similarly effective in the treatment of APN, regardless of whether APN was considered complicated or uncomplicated [29]. This study showed no difference between the carbapenem and amikacin groups in terms of having at least one comorbidity other than urological disease and an underlying urological disease.

In a study by Lee, patients were divided into three groups based on the antimicrobial therapy they received: carbapenem, non-carbapenem, and switched treatment. Comparisons of a prior history of UTIs, previous antibiotic use, and underlying disease revealed no significant differences between the groups [31]. Similarly, this study found no difference between the amikacin and carbapenem groups.

Several studies have underscored the clinical safety of amikacin in pediatric UTIs due to ESBL-positive pathogens. Meltem et al. reported that short-course amikacin therapy was effective and did not result in nephrotoxicity in children when appropriate dosing and monitoring were applied [17]. Similarly, Zohar et al. found no significant renal adverse effects in pediatric patients treated with amikacin for febrile UTIs [18]. In compliance with these reports, no amikacin-induced nephrotoxicity was observed among our study group. This finding emphasizes the idea that amikacin may be a reliable alternative to carbapenem.

Although no statistically significant differences were observed between the carbapenem and amikacin groups in terms of clinical outcomes, the observed trends in clinical improvement and absence of recurrence in both groups are clinically meaningful. The lack of statistical significance may be attributable to the limited sample size, reducing the statistical power of the study. Further multicenter prospective studies with larger populations are required to validate these findings and better assess the non-inferiority of amikacin in this context.

While our study is retrospective and based on clinical data, future experimental studies could provide further insights into the comparative effects of carbapenem and amikacin on bacterial cytotoxicity and host immune interactions. For example, Pastore et al. investigated the antiproliferative properties of Bifidobacterium longum BB-536 in tumor cell lines co-cultured with murine splenocytes and demonstrated that certain probiotic bacterial strains could modulate immune responses and affect cell viability through cytokine-mediated mechanisms [32]. Although the bacterial strains and study context differ, the methodology used—particularly the co-culture model and viability assays (e.g., Trypan Blue exclusion)—may serve as a model for testing the cellular impact of antibiotic therapies on host–pathogen interactions in vitro. Incorporating such experimental approaches could enrich the understanding of how amikacin or carbapenems influence host immune modulation beyond their antimicrobial activity.

## 5. Conclusions

In this retrospective study, no significant difference was observed between carbapenem and amikacin in terms of clinical outcomes, recurrence of bacteriuria, or association with underlying diseases among pediatric patients with ESBL-positive acute pyelonephritis. These findings suggest that aminoglycosides, particularly amikacin, may be a potential alternative to carbapenems in selected pediatric cases.

However, due to the study’s small sample size, retrospective design, and lack of standardized clinical severity assessment, these results should be interpreted with caution. Further prospective studies with larger cohorts are needed to confirm these findings and to support more definitive treatment recommendations for ESBL-related pediatric UTIs.

### Limitations

This study has several limitations that should be considered when interpreting the findings. It was conducted retrospectively at a single tertiary care center with a relatively small patient cohort, which limits the generalizability of the clinical and microbiological results. The absence of a comparison group consisting of patients with APN due to non-ESBL-positive Enterobacteriaceae hinders a clear evaluation of the specific impact of ESBL production on clinical outcomes. Future studies incorporating such control groups are necessary to clarify this issue. Additionally, clinical severity at the time of admission was not systematically recorded, which reflects a common limitation inherent to retrospective study designs.

Another important constraint is the lack of standardized monitoring for amikacin-associated ototoxicity; audiological evaluations were not performed. To comprehensively assess the safety of aminoglycosides in pediatric patients, prospective research with formal hearing assessments is warranted.

## Figures and Tables

**Table 1:** Patient characteristics/demographic data and risk factors.

Characteristic.	Carbapenem Group (n = 20)	Amikacin Group (n = 35)	*p* Value	Total (n = 55)
Age (years)	4.5 (0.1–13)	2 (0.1–7.5)	0.089 †	3 (0.1–13)
Sex			0.895 ‡	
Female	12 (60%)	23 (65.7%)		35 (63.6%)
Male	8 (40%)	12 (34.3%)		20 (36.4%)
Underlying urological disease *	11 (55%)	13 (37.1%)	0.203 ‡	24 (43.6%)
At least one comorbid disease **	4 (20%)	5 (14.3%)	0.585	9 (16.4%)
Antibiotic consumption in last 6 months	8 (40%)	13 (37.1%)	0.346 ‡	28 (50.9%)
UTI in the last year	7 (35%)	18 (51.4%)	0.370 ‡	25 (45.5%)
Etiologic agent				
*E. coli*	15 (75%)	28 (80%)	0.740 †	43 (78.2%)
*Klebsiella* spp.	5 (25%)	7 (20%)		12 (21.8%)
Time to fever reduction				
No fever	1 (5%)	3 (8.6%)		4 (7.3%)
<24 h	13 (65%)	20 (57.1%)	0.775 ‡	33 (60%)
24–48 h	5 (25%)	11 (31.4%)	0.844 ‡	16 (29.1%)
>48 h	1 (5%)	1 (2.9%)	>0.999 †	2 (3.6%)
Time to resolution of pyuria				
<48 h	20 (100%)	34 (97.1%)	>0.999 †	54 (98.2%)
>48 h	0 (0%)	1 (2.9%)		1 (2.8 %)
Clinical improvement	20 (100%)	34 (97.1%)	>0.999 †	54 (98.2%)
Relapse/Recurrence	0 (0%)	0 (0%)	-	0 (0%)

† Fisher’s exact probability test; ‡ chi-square test with continuity correction. UTI: urinary tract infection; * underlying urological diseases included vesicoureteral reflux, ureteropelvic stenosis, neurogenic bladder, chronic kidney disease, urinary stone, renal agenesis, hydronephrosis, and hypospadias; ** comorbid diseases included intestinal lymphangiectasia, cystic fibrosis, trisomy 18, biliary atresia, chronic hepatitis, and systemic lupus erythematosus.

## Data Availability

The data presented in this study are available on request from the corresponding author due to in case of need.

## Abbreviations

The following abbreviations are used in this manuscript:

APN	Acute pyelonephritis
ESBL	Extended-spectrum β-lactamase
UTIs	Urinary tract infections
CFU	Colony-forming units
WBC/HPF	White blood cells per high-power field
MALDI-TOF	Matrix-assisted laser desorption ionization–time of flight
MIC	Minimum inhibitory concentration
EUCAST	European Committee on Antimicrobial Susceptibility Testing
